# Association between sedentary behavior, physical activity, and osteoarthritis: results from NHANES 2007–2020 and Mendelian randomization analysis

**DOI:** 10.3389/fpubh.2024.1454185

**Published:** 2025-01-13

**Authors:** Jiangqin He, Cao Zhang, Lili Yang

**Affiliations:** ^1^Department of Nursing, The Fourth Affiliated Hospital of School of Medicine, and International School of Medicine, International Institutes of Medicine, Zhejiang University, Yiwu, China; ^2^Department of Anesthesia, The Fourth Affiliated Hospital of School of Medicine, and International School of Medicine, International Institutes of Medicine, Zhejiang University, Yiwu, China

**Keywords:** osteoarthritis, sedentary behavior, physical activity, Mendelian randomization, NHANES

## Abstract

**Introduction:**

Osteoarthritis (OA) is a prevalent and debilitating disorder that affects the joints and has a complex array of causes. While sedentary behavior (SB) and physical activity (PA) have been implicated in OA risk, the relationship between these factors and OA development remains unclear. This study investigates the correlation and potential causality between SB, PA, and OA using both cross-sectional and Mendelian randomization (MR) analysis.

**Methods:**

We conducted a two-phase study that included a cross-sectional analysis using data from the National Health and Nutrition Examination Survey (NHANES) and a MR analysis. A weighted analysis was performed on data from the NHANES to explore the relationship between SB, PA, and the risk of OA. Logistic regression was used to assess the association between SB, PA, and OA, adjusting for potential confounders. Non-parametric curve fitting was applied to examine the dose-response relationship between PA levels and OA onset. Additionally, MR was utilized to infer the genetic causality between SB, PA, and OA risk, using genetic instruments as proxies for SB and PA.

**Results:**

The final analysis, which included 24,738 participants, revealed that OA prevalence was 13.47%, with individuals diagnosed with OA exhibiting significantly higher levels of SB and lower levels of PA. A U-shaped relationship was observed between PA and OA risk, with a decrease in OA incidence as PA levels increased, although the protective effect of PA was less pronounced in individuals with severe SB. MR analysis indicated that genetically inferred SB was associated with a higher likelihood of OA (IVW OR 1.20, 95% CI 1.13–1.28), while increased PA was inversely associated with OA risk (IVW OR 0.85, 95% CI 0.73–0.98).

**Conclusion:**

This research emphasizes the significance of SB and PA as modifiable factors influencing the risk of OA. It is recommended that individuals at risk of OA should aim to participate in regular physical activity and minimize sedentary behavior to lower their risk of developing the disease. The MR analysis results support the potential causal impact of SB and PA on OA, providing valuable information for the development of therapeutic and rehabilitative strategies.

## 1 Introduction

Osteoarthritis (OA) is a prevalent and debilitating condition that imposes a substantial health burden (1). The global prevalence of OA has surged by 113.25% from 247.51 million cases in 1990 to 527.81 million cases in 2019, primarily due to the growth of older adult population worldwide (2). The etiology of primary OA is multifactorial, involving genetic predispositions, dietary factors, hormonal influences, and bone density, among other elements (3).

While non-pharmacological interventions such as weight management, regular physical activity, and trauma prevention are widely recognized in the management and prevention of OA (4), the role of sedentary behavior (SB) has increasingly come under scrutiny. SB is defined as activities with minimal increases in energy expenditure, typically ≤1.5 metabolic equivalents (METs). These activities include screen time, driving, reading, and studying, and have been identified as independent health risk factors in contemporary society (5).

The relationship between physical activity (PA) and OA is also a subject of ongoing research. While high levels of PA are generally associated with health benefits, their role in the incidence and progression of OA is less clear. Some studies have indicated that high-intensity PA may increase the risk of OA, particularly knee OA (6, 7), while moderate-intensity PA may offer protective benefits (8).

Recent evidence suggests that SB may contribute to the development of OA, potentially through its association with increased body mass index and obesity, which are established risk factors for the condition (9). However, the relationship between SB and OA is complex and not yet fully elucidated. Some studies have reported no significant difference in SB between individuals with and without knee OA (10), while others have found that reducing SB duration can improve pain and symptoms in those diagnosed with OA (11). Furthermore, in a 4-year follow-up study of 1,091 individuals, Master et al. conducted a comprehensive analysis by combining SB and PA. The results showed that individuals classified as inactive high sedentary had a 52% higher risk of developing osteoarthritis compared to those classified as active low Sedentary (12).

Given the limitations of existing observational studies, such as small sample sizes and insufficient adjustment for significant covariates, the current study aims to leverage the National Health and Nutrition Examination Survey (NHANES) database and Mendelian randomization (MR) analysis to investigate the association between sedentary behavior, physical activity, and osteoarthritis. Mendelian randomization (MR) analysis is a tool that uses genetic data to explore causal relationships between exposures and outcomes (13), that are less susceptible to confounding biases and reverse causal risk because gene alleles are randomly assigned during meiosis and are independent of environmental factors (14). Therefore, MR studies are commonly referred to as “randomized, double-blind trials created by nature” and are considered complementary to randomized controlled trials (RCTs). Due to the inconsistent results of observational studies and the lack of robust evidence from RCTs, MR analysis can be a useful tool for exploring the causal relationship between SB, PA, and osteoarthritis.

Based on previous studies, we hypothesize that both SB and PA contribute to OA and that an interaction exists between these factors in influencing OA risk. We utilize data from the NHANES database and Mendelian randomization analysis to explore this interaction and better understand its impact. This approach allows us to mitigate the limitations associated with confounding bias and reverse causation, providing more robust insights into the relationship between SB and PA with OA.

## 2 Materials and methods

### 2.1 Overall study design

**Figure 1:**
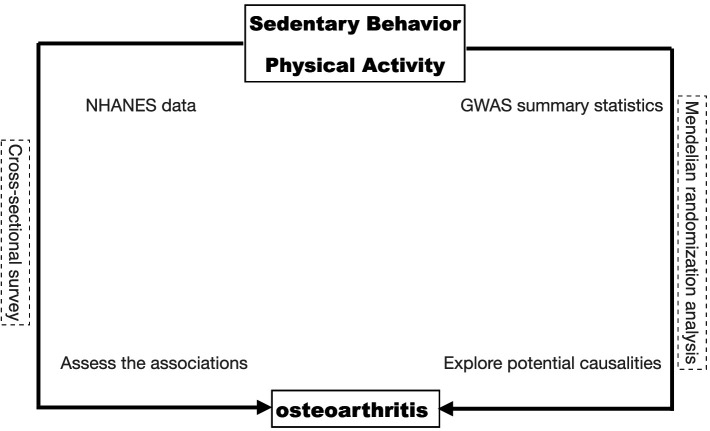
The overall design of the current study.

This study was conducted in two stages; first using the data deposited in the National Health and Nutrition Examination Survey (NHANES) database, we performed a variety of statistical methods to explore the association between sedentary behavior, physical activity, and osteoarthritis. In stage 2, we assessed the causal effect of genetically determined SB and PA on osteoarthritis by MR analysis of summary statistics data from the genome-wide association study (GWAS). The overall study design is shown in Figure 1.

### 2.2 Data sources and study population

The study used data from the National Health and Nutrition Examination Survey (NHANES), a population survey conducted by the Centers for Disease Control and Prevention (CDC) in the United States. The NHANES has collected data since 1999 through 2-year cycles and aims to gather information from approximately 10,000 individuals. The survey includes interviews, physical examinations, and laboratory tests. A multistage probability sampling design was used to select a sample of non-institutionalized households across the country to ensure representativeness. All participants provided written informed consent before participating. The public data and survey design are available on the NHANES website.

**Figure 2:**
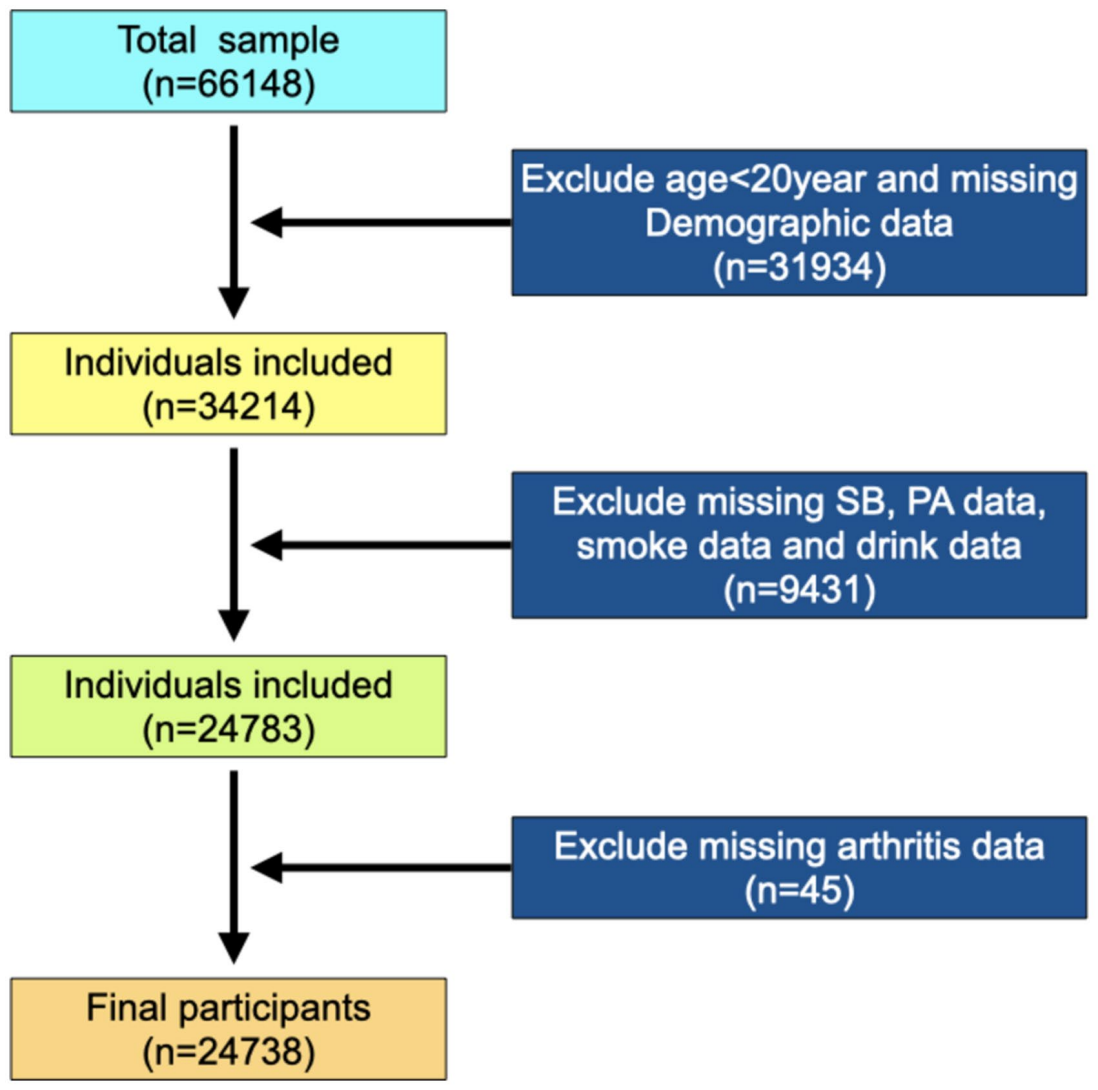
Flowchart of the study design and participants excluded from the study.

The present study population was from six cycles of “continuous NHANES” (2007/2008, 2009/2010, 2011/2012, 2013/2014, 2015/2016, and 2017/2020). A total of 66,148 participants were included in the analysis, excluding participants younger than 20 and missing demographic data (*n* = 31,934). Subsequently, eligible participants needed complete data on SB, PA, and osteoarthritis report data. This resulted in an analytic sample of 24,738 survey participants (Figure 2).

### 2.3 Measurement of sedentary behavior and physical activity

The NHANES study assessed physical activity levels and sedentary time were using the self-administered Physical Activity Questionnaire (PAQ). The PAQ consists of 18 questions, and previous studies have demonstrated that PAQ has moderate to good test–retest reliability (15, 16). Within this questionnaire, sedentary behavior was ascertained by asking participants about the time spent sitting at school, at home, getting to and from places, or with friends including time spent sitting at a desk, traveling in a car or bus, reading, playing cards, watching television, or using a computer throughout a typical day. In accordance with recent literature, the daily sitting time for cancer survivors in this study was categorized into predefined thresholds: less than 8 h and more than 8 h (12). The self-reported physical activity (PA) was collected through questionnaires that asked about the frequency, duration, and intensity of physical activity, including moderate and vigorous activity. The suggested metabolic equivalent (MET) scores for the activities were 4 MET for moderate activity and 8 MET for vigorous activity. Participants were deemed “active” if their reported physical activity adhered to the World Health Organization’s (WHO) guidelines, which are as follows: a minimum of 150–300 min/week of moderate-intensity aerobic activity, or a minimum of 75–150 min of vigorous-intensity aerobic activity, or a commensurate blend of both intensities. Individuals not meeting these criteria were labeled “inactive” (17).

### 2.4 Assessment of osteoarthritis

The questionnaire assessed the OA status of the participants. All participants were asked whether they had ever been diagnosed with arthritis: “Has a doctor or other health professionals ever told you that you had arthritis?” If participants answered “Yes,” then they were asked, “Which type of arthritis was it?” According to the answers to these questions, participants were defined as having OA or no OA.

### 2.5 Data collection

Several variables for all participants were extracted. Information was obtained from self-reported data, including age (years), sex, ethnicity, education level, marital status, alcohol consumption, smoking, physical activity (MET·h), and history of OA. Body mass index (BMI, kg/m^2^) was obtained from examination data. BMI was calculated as weight (kg) divided by height squared (m^2^). Physical activity (MET·h) was calculated as a MET according to the compendium of physical activities = MET × exercise time of corresponding activity (h/week).

### 2.6 Statistical analysis

In view of the complex multistage (strata and cluster) sampling design of the NHANES, the survery package(version 4.2) of R was used to conduct the weighted analysis. Sample weights from the MEC interviews were reweighted to merge 7.2 years of total survey data from the NHANES 2013–2020. The merged weights were represented as WT = (2/13.2) × (WTMEC2YR07–08) + (2/13.2) × (WTMEC2YR09–10) + (2/13.2) × (WTMEC2YR11–12) + (2/13.2) × (WTMEC2YR13–14) + (2/7.2) × (WTMEC2YR15–16) + (3.2/13.2) × WTMECPRP.

For this study, we utilized different statistical methods based on the distribution and nature of the data. Measurement data with a normal distribution were presented as mean (standard error) (SE), and a comparison between groups was conducted using the independent sample *t*-test. On the other hand, measurement data with a non-normal distribution were described using the median and quartile spacing [M(Q1, Q3)], and a comparison between groups was performed using the Mann–Whitney U rank–sum test. Enumeration data were presented as the number of cases and composition ratio n (%). The chi-square test was used to compare groups for enumeration data, while the rank-sum test was used for rank data. All statistical tests were conducted using a two-sided approach, and a significance level of *p* < 0.05 was used to determine statistical significance. The statistical analysis was performed using R software^1^.

To explore the relationship between SB and PA with the prevalence of OA, we employed the locally weighted scatterplot smoothing (LOWESS) method for non-parametric curve fitting. LOWESS is a flexible non-parametric regression technique that creates a smooth curve by assigning different weights to each data point, allowing us to observe potential trends in the data without the strict assumption of linearity. In R, we utilized the ggplot2 package^2^ to generate the graphical representation.

### 2.7 Two-sample Mendelian randomization

Summary-level results from the genome-wide association studies (GWAS) were used in this study, including subjectively assessed SB with leisure screen time and to be used as exposures were obtained from the GWAS Catalog. All data in the GWAS Catalog are de-identified and openly available for research purposes. This SB study embodied the European population sample and the whole-genome sequencing data of SB (*n* = 526,725) (18). The PA exposure data were also from the GWAS Catalog (*n* = 88,411) assessed PA by total log acceleration (19). The outcome data were OA from the GWAS Catalog (*n* = 456,348) (20).

For the univariable Mendelian randomization (UVMR), all genome-wide significant single nucleotide polymorphisms (SNPs) were filtered out with a strict threshold of *p* < 5 × 10^−7^ and were then clumped with a cutoff of *r*^2^ = 0.001 and kb = 10,000 for the avoidance linkage disequilibrium (LD). The SNPs in the selection were also matched to the databases of phenome-wide association studies to prevent the underlying link between the SNPs and confounding factors by limiting the *p* < 5 × 10^−6^ threshold. We also harmonized exposures and outcomes regarding effect allele and applied the subsequent analysis based on the combined exposure–outcome dataset. Detailed information on IVs is shown in Supplementary Tables S1, S2.

R packages TwoSampleMR^3^ (version 0.5.8) was used to implement five MR methods (inverse variance weighted [IVW], MR-Egger, weighted mode, weighted median, and simple mode) under three basic assumptions in the univariable MR. We estimated the causal effects of genetically predicted exposure on the outcome using the IVW method as our primary MR analysis method, the IVW adopted a meta-analytical approach to combine Wald estimates of causality for each instrumental variable and to obtain a stable causal estimate with balanced pleiotropy. Additionally, the MR-Egger regression and weighted median method were implemented in addition to the IVW, since these methods can provide more comprehensive estimates. To address multiple hypothesis testing, we estimated the false discovery rate (FDR) adjusted *p*-values (*q*-values) in the primary IVW MR analyses using the sequential *p*-value approach proposed by Benjamini and Hochberg.

## 3 Results

### 3.1 Baseline characteristics

There were 24,738 participants eligible for our final analysis, and the weighted number of participants was 162,563,145, and 2,604 osteoarthritis patients were included in the study cohort. The weighted prevalence of osteoarthritis within the study population was 13.47%, corresponding to a total of 23,168,508 individuals. The weighted mean daily sitting time was 385.93 ± 3.39 min per day, and the weighted mean physical activity was 70.07 ± 1.74 MET/week. The weighted mean age of the participants was 46.84 ± 0.27 years, and 48.39% were men. Among these participants, 15,857,229 (67.61%) were non-Hispanic white, 560,143 (8.09%) were Mexican American, 575,217 (5.84%) were other Hispanic, 1,174,222 (10.72%) were non-Hispanic Black, and 927,974 (7.73%) were other ethnic group populations (Table 1). Additionally, the weighted prevalence of osteoarthritis by age, sex, ethnicity, body mass index, marital status, education, smoking status, and alcohol consumption status was statistically significantly different (*p* < 0.001). However, no significant difference was observed between the incidence of osteoarthritis in relation to the severity of SB (mild vs. severe). Notably, there was a discernible difference in SB over time and in the levels of PA as measured by MET/week.

**Table 1:** Weighted characteristics of the study population by osteoarthritis status.

Characteristics	Participants % (SE)	Osteoarthritis % (SE)	Non-osteoarthritis % (SE)	*p*-value
Age (years)				<0.001
<44	46.82 (0.71)	10.69 (0.70)	51.63 (0.71)	
[44, 60)	27.97 (0.46)	29.80 (1.08)	27.73 (0.50)	
[60, 75)	18.72 (0.52)	42.60 (1.31)	15.54 (0.49)	
≥75	6.49 (0.23)	16.92 (0.78)	5.10 (0.21)	
Sex				<0.001
Male	48.39 (0.37)	33.58 (1.21)	50.35 (0.39)	
Female	51.62 (0.37)	66.41 (1.21)	49.65 (0.39)	
Ethnicity				<0.001
Non-Hispanic White	67.61 (1.37)	83.05 (1.03)	65.56 (1.42)	
Mexican American	8.09 (0.67)	2.93 (0.37)	8.78 (0.72)	
Other Hispanic	5.84 (0.46)	3.01 (0.32)	6.22 (0.49)	
Non-Hispanic Black	10.72 (0.70)	6.15 (0.59)	11.33 (0.74)	
Other ethnicity	7.73 (0.41)	4.86 (0.57)	8.11 (0.43)	
Education				0.036
Below high school	13.58 (0.54)	10.46 (0.73)	13.99 (0.55)	
High school	23.20 (0.61)	23.41 (1.28)	23.17 (0.68)	
College or above	63.23 (0.93)	66.13 (1.41)	62.84 (0.98)	
Marital status				<0.001
Married/living with Partner	63.18 (0.73)	65.18 (1.34)	62.91 (0.76)	
Widowed/divorced	22.86 (0.58)	30.07 (1.24)	21.9 (0.59)	
Never married	13.96 (0.57)	4.75 (0.58)	15.19 (0.62)	
Poverty income ratio				0.0013
<1.3	14.01 (0.56)	9.66 (0.79)	14.59 (0.58)	
[1.3, 3.5)	34.87 (0.74)	34.75 (1.46)	34.89 (0.76)	
≥3.5	51.12 (0.96)	55.59 (1.83)	50.52 (0.99)	
BMI				<0.001
<18.5	1.52 (0.10)	0.77 (0.19)	1.62 (0.11)	
[18.5, 25)	27.70 (0.51)	20.55 (1.12)	28.65 (0.56)	
[25, 30)	32.69 (0.43)	31.78 (1.21)	32.81 (0.44)	
≥30	38.09 (0.56)	46.90 (1.31)	36.92 (0.60)	
Smoke				<0.001
Never smoker	56.85 (0.63)	48.9 (1.49)	57.91 (0.68)	
Former smoker	23.95 (0.50)	35.22 (1.45)	22.45 (0.5)	
Current smoker	19.20 (0.50)	15.88 (1.03)	19.64 (0.54)	
Alcohol consumers (Number of alcohol consumption/week)				<0.001
0	25.31 (0.66)	30.11 (1.42)	24.67 (0.65)	
<1	22.86 (0.45)	27.75 (1.46)	22.21 (0.50)	
[1,8]	35.87 (0.52)	29.06 (1.25)	36.78 (0.54)	
>8	15.96 (0.42)	13.08 (0.91)	16.34 (0.45)	
Sedentary behavior time (minute)	371.43 ± 2.76	386.21 ± 4.78	369.46 ± 2.96	0.002
Sedentary behavior				0.655
Mild (<8 h/day)	77.00 (0.53)	76.58 (1.01)	77.06 (0.56)	
Severe (≥8 h/day)	23.00 (0.53)	23.42 (1.01)	22.94 (0.56)	
Physical activity (MET × h/week)	66.49 ± 1.19	47.30 ± 2.27	69.05 ± 1.25	<0.001
Physical activity				<0.001
Inactive	61.03 (0.56)	70.81 (1.19)	59.73 (0.59)	
Active	38.97 (0.56)	29.19 (1.19)	40.27 (0.59)	

### 3.2 Logistic regression

**Figure 3:**
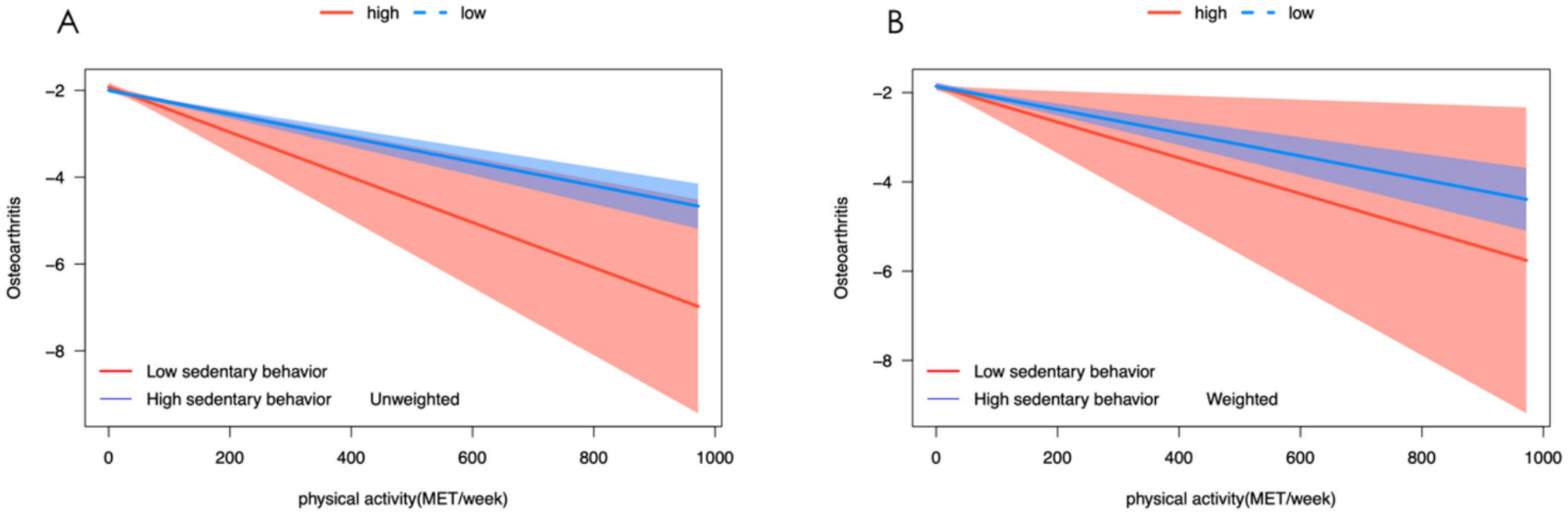
Association between sedentary behavior, physical activity, and osteoarthritis. **(A)** Unweighted logistic model to predict the occurrence of osteoarthritis and levels of physical activity in varying degrees of sedentary behavior. **(B)** Weight logistic model to predict the occurrence of osteoarthritis and levels of physical activity in varying degrees of sedentary behavior.

We conducted both unweighted and weighted logistic regression analyses to assess the interaction between SB and PA in relation to the incidence of OA. The results in Table 2 and Figure 3 elucidate the complex relationship between SB, PA, and the risk of developing OA. The incidence of osteoarthritis decreased with the increase of PA [unweighted OR: 0. 9,971 (0.9966, 0.9976), weighted OR: 0.9974 (0.9966, 0.9981)]. The interaction between SB and PA showed that PA had a more significant impact on the incidence of osteoarthritis when individuals were in severe SB, although there was no statistical difference.

**Table 2:** The Logistic Regression analyses to assess the interaction between SB and PA in relation to the incidence of OA.

	Unweighted OR	*p*-value	Weighted OR	*p*-value
Mild SB	Reference		Reference	
Severe SB	1.1250 (1.0176, 1.2420)	0.020*	1.0275 (0.9114, 1.1583)	0.655
Physical activity	0.9971 (0.9966, 0.9976)	<0.001*	0.9974 (0.9966, 0.9981)	<0.001*
Physical activity × mild SB	0.9972 (0.9966, 0.9977)	<0.001*	0.9974 (0.9966, 0.9982)	<0.001*
Physical activity × severe SB	0.9955 (0.9932, 0.9977)	<0.001*	0.9960 (0.9924, 0.9996)	0.029*
Active group	Reference		Reference	
Inactive group	1.6020 (1.4635, 1.7554)	<0.001*	1. 6,357 (1.4510, 1.8439)	<0.001*
SB time	1.0007 (1.0005, 1.0009)	<0.001*	1.0004 (1.0002, 1.0006)	0.002*
SB time × Active group	0.9997 (0. 9,994, 0.9999)	0.044*	0. 9,993 (0.9990, 0.9997)	0.001*
SB time × Inactive group	1.0008 (1.0007, 1.0011)	<0.001*	1.0006 (1.0003, 1.0008)	<0.001*

SB: sedentary behavior.

Physical activity × SB: interaction between physical activity and sedentary behavior.

SB time × active or inactive group: interaction between sedentary behavior and different degrees of physical activity.

^*^p < 0.05.

### 3.3 Non-parametric curve fitting

**Figure 4:**
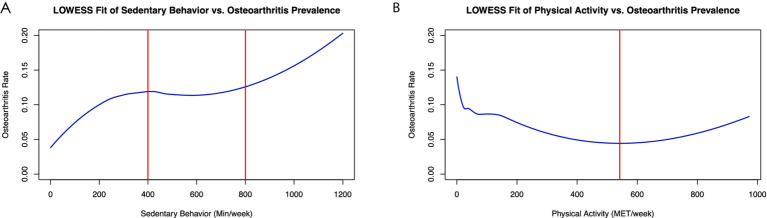
Association between sedentary behavior, physical activity, and osteoarthritis. **(A)** Shows a positive correlation between weekly minutes of sedentary behavior and osteoarthritis incidence using a LOWESS fit. A plateau in the relationship is observed between 400 and 800 min of inactivity per week. **(B)** Depicts a “U-shaped” trend between physical activity (MET/week) and osteoarthritis incidence, as fitted by LOWESS. The lowest osteoarthritis rate is found with a 550 MET/week physical activity level.

We employed the LOWESS method for non-parametric curve fitting to elucidate the relationship between SB, PA, and the incidence of OA. As depicted in Figure 4, our analysis revealed a positive correlation between the prevalence of OA and the duration of SB in general. However, this correlation was attenuated within the moderate range of 400–800 min of sedentary activity per week, suggesting a more complex relationship within this specific interval. Concurrently, the relationship between PA and OA exhibited a “U-shaped” trend, indicating that very low and very high levels of exercise could be associated with higher rates of OA. Interestingly, the incidence of osteoarthritis reached its nadir when the weekly exercise equivalent was approximately 550 MET·h/week.

**Figure 5:**
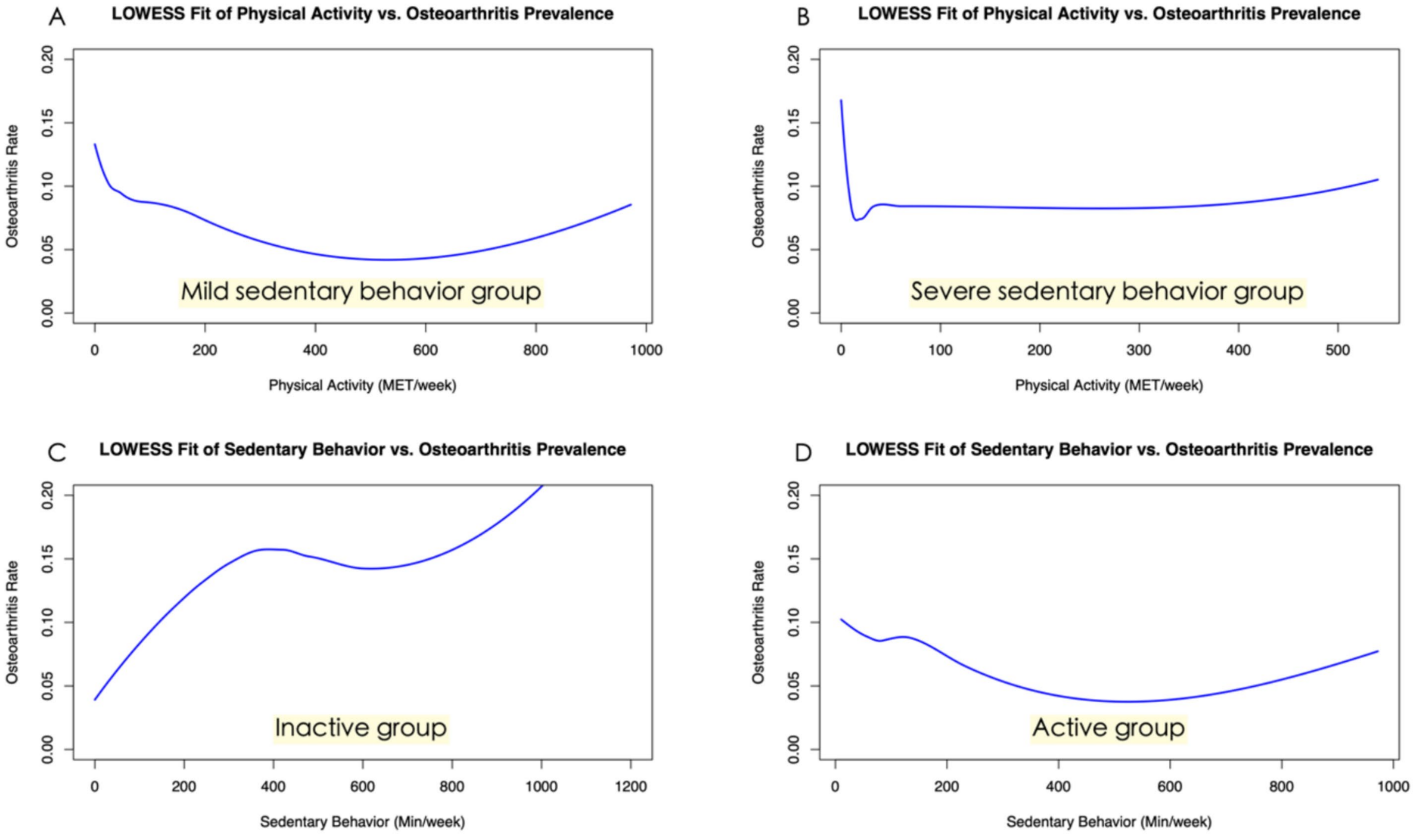
Subgroup analysis of the association between sedentary behavior, physical activity, and osteoarthritis. **(A)** This panel illustrates the relationship between physical activity and osteoarthritis prevalence among participants with mild sedentary behavior. **(B)** The “L-shaped” trend in this figure indicates the correlation between physical activity and osteoarthritis prevalence within the severe sedentary behavior group. **(C)** Shown here is the robust positive correlation between sedentary behavior and osteoarthritis prevalence in the inactive subgroup, highlighting the risks of inactivity. **(D)** This figure illustrates a weaker link between sedentary behavior and osteoarthritis prevalence in the active subgroup.

In a detailed subgroup analysis, we stratified participants by the severity of their sedentary behavior into severe and mild groups (Figures 5A,B). Notably, physical exercise incidence vs. osteoarthritis within the severe group presented an “L-shaped” trend (Figure 5B). Further categorization by PA intensity into active and inactive groups revealed contrasting correlations using LOWESS curve fitting; active individuals showed a weaker link between sedentary behavior and osteoarthritis (Figure 5D), while the inactive demonstrated a robust positive correlation (Figure 5C), underscoring the protective role of PA against the detrimental effects of SB.

### 3.4 Two-sample Mendelian randomization of sedentary behavior and physical activity attainment on osteoarthritis

**Figure 6:**
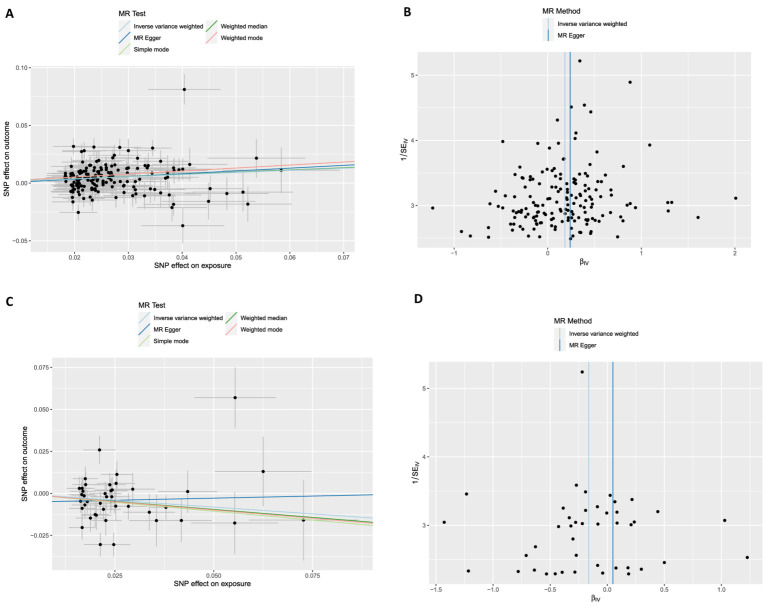
Scatter plots and funnel plots from genetically predicted physical activity and sedentary behavior on osteoarthritis and severity. **(A,B)** Genetically predicted sedentary behavior on osteoarthritis; **(C,D)** Genetically predicted physical activity on osteoarthritis.

The number of instrumental variables for SB on osteoarthritis was 169 (Supplementary Table S1). The IVW analysis indicated that the accelerometer assessed SB increased the risk for osteoarthritis (OR = 1.20, 95% CI: 1.13–1.28; FDR < 0.001; *p* < 0.001). Similar causal estimates for osteoarthritis were obtained from other MR models, including weighted median (OR = 1.21, 95% CI: 1.12–1.30; FDR < 0.001; *p* < 0.001), and simple model (OR = 1.30, 95% CI: 1.03–1.64; FDR = 0.052; *p* = 0.031) (Table 3; Figure 6). Genetically proxied physical activity was significantly associated with a decreased risk of osteoarthritis, as in the IVW analysis model (OR = 0.85, 95% CI: 0.73–0.98; FDR = 0.052; *p* = 0.029), and Weighted median (OR = 0.83, 95% CI: 0.71–0.96; FDR = 0.038; *p* = 0.011) (Table 3). The scatter plots and funnel plots (Figure 6) showed a significant causal relationship between SB, PA, and OA.

**Table 3:** Two-sample Mendelian randomization of sedentary behavior and physical activity attainment on osteoarthritis.

Exposure outcome	Sample size	OR	*p*-value
SB – Osteoarthritis	169		
MR Egger		1.27 (0.95, 1.7)	0.10995
Weighted median		1.21 (1.12, 1.3)	0.00000*
Inverse variance weighted		1.20 (1.13, 1.28)	0.00000*
Simple mode		1.30 (1.03, 1.64)	0.03094*
Weighted mode		1.30 (1.05, 1.6)	0.01574*
PA – Osteoarthritis	43		
MR Egger		1.05 (0.65, 1.69)	0.83859
Weighted median		0.83 (0.71, 0.96)	0.01144*
Inverse variance weighted		0.85 (0.73, 0.98)	0.02945*
Simple mode		0.81 (0.61, 1.08)	0.15327
Weighted mode		0.82 (0.65, 1.04)	0.10782

SB, sedentary behavior; PA, physical activity.

^*^p < 0.05.

## 4 Discussion

To the best of our knowledge, this study is the first comprehensive investigation of the relationship between sedentary behavior and physical activity with osteoarthritis based on large-scale observational research data (NHANES dataset) and MR analysis of large-scale genetic data (GWAS). We examined the association between PA and SB with OA. Furthermore, we investigated the potential causal relationship between exercise lifestyle and OA. In our analysis of a large national sample (weighted number 162,563,145), we observed that participants with osteoarthritis exhibited significantly higher levels of sedentary behavior and lower physical activity. The incidence of osteoarthritis decreased with the increasing MET scores but was higher among individuals with severe sedentary behaviors. However, no significant difference was found between mild (<8 h/day) and severe (8 h/day or more) SB in the OA difference group. The non-parametric curve fitting analysis, similar to a logistic model, indicates a positive correlation between sedentary behavior and the prevalence of osteoarthritis. We observed a U-shaped relationship between the volume of physical activity and osteoarthritis development. Additionally, the subgroup analysis revealed that individuals who engaged in less than 50 METs of activity per week exhibited a significant increase in osteoarthritis within the group characterized by severe sedentariness. Furthermore, the adverse impact of sedentary behavior on osteoarthritis risk was considerably reduced in individuals who adhered to the WHO-recommended standards for weekly physical activity. The two-sample Mendelian randomization analysis results suggest that physical activity may reduce the risk of osteoarthritis. In contrast, sedentary behaviors may have the opposite effect. Additionally, the protective effect of physical activity on osteoarthritis was weakened in the group with severe sedentary behaviors, as found by subgroup analysis in both mild and severe sedentary behaviors groups.

While previous studies have investigated the relationship between sedentary behavior (SB) and osteoarthritis (OA), our analysis offers a novel perspective on this association. Using the NHANES dataset, we found that participants with OA exhibited significantly higher levels of sedentary behavior. However, no significant differences were observed between the mild and severe SB groups, categorized based on whether participants spent more or less than 8 h per day in sedentary activities. This lack of distinction may be explained by the non-linear effect of sedentary behavior on OA. The curve fitted from the NHANES data suggests that sedentary behavior has little impact on the incidence of OA when its duration falls within the range of 400–800 min/week. The two-sample Mendelian randomization analysis identified SB as a risk factor for OA (IVW OR = 1.2; weighted median OR = 1.21), the same as the result of Cao’s Mendelian randomization analysis (21). The increased risk of OA may be attributed to the loss of articular cartilage. This was confirmed in Nomura’s animal study, where the total amount and thickness of cartilage in the hind limbs of mice were significantly reduced by passively lowering the weight on the hind limbs (22).

Previous studies have indicated that the relationship between PA and OA remains a topic of contention (23). Recent research studies have shown that PA could reduce pain and improve function in OA patients since PA can prevent obesity or lower limb muscle weakness (4, 24, 25). Despite this, there is insufficient research evidence to confirm the role of PA in the development of osteoarthritis. Chang et al. (26) conducted a cohort study following 1,194 participants for over a decade and reported no association between long-term vigorous PA and knee OA. The study also indicated that a low-to-moderate level of PA may have a protective effect on knee OA. A cohort study of healthy adults, defined as minimally active, revealed a 72% greater risk of developing OA (12). This may improve cartilage and potentially prevent OA degeneration (27). Our results from the NHANES data indicate that an increase in weekly physical activity is associated with a reduction in the incidence of osteoarthritis. Interestingly, our analysis also suggests that if the amount of exercise exceeds a certain threshold, its protective effect diminishes. This aligns with the common understanding that excessive physical activity can lead to sports-related injuries, potentially increasing the risk of osteoarthritis. In our study, 550 MET·hour per week emerged as the optimal amount of exercise for reducing osteoarthritis risk. Furthermore, we employed a two-sample Mendelian randomization analysis further to clarify the causal relationship between PA and OA. These findings suggest that PA served as a protective factor against the development of osteoarthritis.

PA and SB are two crucial factors that influence the incidence of OA. Previous studies have tended to focus on only one of these factors. Therefore, it is important to consider the interplay between them. To investigate the impact of SB and PA on osteoarthritis, we conducted a subgroup analysis, dividing it into mild and severe SB groups. The NHANES data revealed that the protective effect of PA on osteoarthritis was weakened among individuals with high levels of SB, regardless of whether weighted analysis was performed. Research conducted by Master et al. also found that high sedentary would increase the risk of OA regardless of one’s PA category (12). Similarly, in the study of other diseases, the effects of PA and SB on these diseases are also linked. Balducci et al. indicated that increasing moderate-to-vigorous physical activity while substantially reducing SB time could provide benefits for patients with type-2 diabetes (28). The biomechanical factors of OA are hypothesized to influence the relationship between PA and SB with OA. Alterations in mechanical loading on joints have been demonstrated to impact cartilage health—potentially contributing to the deterioration of joint tissues (29). Additionally, PA enhanced the metabolism of synovial fluid, which may have implications for the structure of subchondral bone (30).

There were several limitations in our study. First, the observational design of the NHANES dataset restricted our ability to establish causal relationships. Although MR analysis was employed to verify this association, it was constrained by several assumptions. For example, it assumes no pleiotropic association between genetic variants and phenotypes, as well as a linear genetic relationship. Second, the GWAS datasets were derived from large cohorts of European ancestry, whereas the NHANES datasets were collected in the United States. As a result, there was some ethnic diversity, and caution should be exercised when extrapolating the results to other ethnic groups. Rigorously designed randomized controlled trials are warranted to better understand the causal relationship between PA and SB with osteoarthritis.

## 5 Conclusion

Our research suggested that physical activity and sedentary behaviors were significant factors in the prevention of osteoarthritis. Individuals at risk for osteoarthritis should aim to engage in a regular level of physical activity while reducing sedentary behaviors. This research examines the potential causal relationship between physical activity, sedentary behaviors, and osteoarthritis. It may help identify new treatment and rehabilitation strategies to address this condition.

## Data Availability

The datasets presented in this study can be found in online repositories. The names of the repository/repositories and accession number(s) can be found at: https://www.cdc.gov/nchs/nhanes/, https://www.ebi.ac.uk/gwas/.

## Footnotes

^1^ http://www.r-project.org/

^2^ https://cran.rstudio.com/web/packages/ggplot2/index.html

^3^ https://github.com/MRCIEU/TwoSampleMR/releases
